# Identification of aberrant forms of alkaline sphingomyelinase (NPP7) associated with human liver tumorigenesis

**DOI:** 10.1038/sj.bjc.6604013

**Published:** 2007-10-09

**Authors:** Y Cheng, J Wu, E Hertervig, S Lindgren, D Duan, Å Nilsson, R-D Duan

**Affiliations:** 1 Gastroenterology Lab, Biomedical Centre, B11, Institute of Clinical Sciences, Lund University, Lund S-221 84, Sweden; 2 Beijing Institute of Biotechnology, No. 20, Dong Dajie, Beijing 100071, China; 3 Gastroenterology Division, Institute of Clinical Sciences, Lund University, Lund S-221 85, Sweden; 4 Gastroenterology Division, Department of Clinical Sciences, Lund University, Malmö S-20502, Sweden

**Keywords:** alkaline sphingomyelinase, liver tumour, HepG2, liver diseases, human, ENPP7

## Abstract

Alkaline sphingomyelinase (alk-SMase) is expressed in the intestine and human liver. It may inhibit colonic tumorigenesis, and loss of function mutations have been identified in human colon cancer. The present study investigates its expression in human liver cancer. In HepG2 liver cancer cells, RT–PCR identified three transcripts with 1.4, 1.2 and 0.4 kb, respectively. The 1.4 kb form is the wild-type cDNA with five translated exons, the 1.2 kb product lacks exon 4 and the 0.4 kb form is a combination of exons 1 and 5. Genomic sequence showed that these aberrant transcripts were products of alternative splicing. Transient expression of the 1.2 kb form showed no alk-SMase activity. In HepG2 cells, the alk-SMase activity is low in monolayer condition and increased with cell polarisation. Coexistence of 1.4 and 1.2 kb forms was also identified in one hepatoma biopsy. GenBank search identified a cDNA clone from human liver tumour, which codes a protein containing full length of alk-SMase plus a 73-amino-acid tag at the N terminus. The aberrant form was translated by an alternative starting codon upstream of the wild-type mRNA. Expression study showed that linking the tag markedly reduced the enzyme activity. We also analysed human liver biopsy samples and found relatively low alk-SMase activity in diseases with increased risk of liver tumorigenesis. In conclusion, expression of alk-SMase is changed in hepatic tumorigenesis, resulting in loss or marked reduction of the enzyme function.

Alkaline sphingomyelinase (alk-SMase), discovered in 1969, hydrolyses sphingomyelin (SM) at optimal alkaline pH (Nilsson, 1969). Recent cloning studies showed that the enzyme shares no similarities with other SMases but belongs to nucleotide pyrophosphatase/phosphodiesterase (NPP) family (Duan *et al*, 2003; Wu *et al*, 2005a). Being a novel member of the NPP family, it is also named NPP7 (Stefan *et al*, 2005). Alk-SMase is an ectoenzyme with a signal peptide at the N terminus and a hydrophobic domain at the C terminus. The N-terminal signal peptide is important for transport of the enzyme to plasma membrane, whereas the C-terminal hydrophobic domain is a signal anchor, which hooks the enzyme to the surface of plasma membrane (Duan *et al*, 2003; Wu *et al*, 2004b). The enzyme activity has been identified in the intestinal tract of many species (Duan *et al*, 1996) and human bile (Nyberg *et al*, 1996; Duan and Nilsson, 1997) but not in bile of other species such as pig, rat, mouse, rabbit, sheep and baboon. In agreement, northern blot has shown high levels of alk-SMase mRNA in human liver (Duan *et al*, 2003) but not in rat liver (Wu *et al*, 2005a). The enzyme appearing in the bile is believed to be released from liver by bile salt (Duan *et al*, 1998). The enzyme in the intestinal mucosa can also be released by trypsin, and the cleaved enzyme has higher activity than the mucosal form (Wu *et al*, 2004b).

Sphingomyelin metabolism generates both antiproliferative molecules such as ceramide and sphingosine and proliferative molecules such as sphingosine-1-phosphate, which are deeply involved in development of many types of tumour (Ogretmen and Hannun, 2004). Alk-SMase may function as a tumour suppressor through three mechanisms, as recently reviewed (Duan, 2006). First, it hydrolyses SM to generate ceramide, which inhibits cell proliferation, and induces apoptosis mainly by dephosphorylation and inactivation of many proliferative and antiapoptotic molecules such as protein kinase B (Akt), protein kinase C, Bcl-2 and pRB (Merrill *et al*, 1997; Pettus *et al*, 2002). Second, it hydrolyses and inactivates platelet-activating factor, a potent proinflammatory and proliferative molecule in the intestinal tract (Wu *et al*, 2006). Finally, it degrades lysophosphatidylcholine by a phospholipase C activity, thus reducing the formation of lysophosphatidic acid, a potent factor that stimulates cell migration and angiogenesis (Duan, 2006; Wu *et al*, 2006). We previously found that alk-SMase activity was decreased in colonic adenomas and carcinomas, in familial adenomatous polyposis and in longstanding ulcerative colitis (Hertervig *et al*, 1997, 1999; Sjöqvist *et al*, 2002). The reduction was recently confirmed and the levels of alk-SMase in the faeces have been considered as a potential marker for diagnosis and prognosis of colon cancer (Di Marzio *et al*, 2005). The activity reduction may be a consequence of genetic perturbations, as an exon deletion that inactivates the enzyme has been found in HT29 colon cancer cells (Wu *et al*, 2004a).

While much attention has been paid to the enzyme in colon, little is known about the changes of the enzyme in human liver, the only organ except intestine that expresses alk-SMase (Duan *et al*, 1996, 2003; Wu *et al*, 2005a). Liver is important for generating endogenous SM and regulating the SM levels in the plasma (Nilsson and Duan, 2006), and previous studies have shown that SM metabolism is implicated in liver diseases. Sphingomyelin has been reported to protect hepatic cells against bile salts (Amigo *et al*, 1999; Moschetta *et al*, 2003). Animals ingesting food contaminated with mycotoxin fumonisin B, an inhibitor of ceramide synthesis, have an increased risk of liver cancer (Merrill *et al*, 1995; Gelderblom *et al*, 1996). Feeding animals with dietary SM reduced the formation of liver enzyme-altered foci, a hepatic marker of preneoplasia (Silins *et al*, 2003). Platelet-activating factor, another substrate for alk-SMase, has important implications in carbon tetrachloride-induced liver cirrhosis (Yang *et al*, 2004), endotoxin- and alcohol-induced hepatic apoptosis and necrosis (Murohisa *et al*, 2002), and liver metastasis of colon cancer (Denizot *et al*, 2005). The present study for the first time examines the expression of alk-SMase in human liver cancer cells and diseased liver tissues. Our results indicate that the expression of alk-SMase can be changed in human liver tumour and liver diseases, resulting in marked reduction of the enzyme activity.

## MATERIALS AND METHODS

### Materials

HepG2 and COS-7 cells were purchased from American Tissue Culture Collection. AK126250 clone was purchased from National Institute of Technology and Evaluation (Chiba, Japan). Anti-human alk-SMase antibody was generated by AgriSera AB (Vännäs, Sweden) as described (Duan *et al*, 2003). Sphingomyelin was labelled with [^14^C-CH_3_]choline ([^14^C-SM]) at Astra Zeneca, (Södertälje, Sweden) (Stoffel, 1975). Plasmid pcDNA4/TO/myc-His B, lipofectamine™ 2000, Ready-To-Go RT–PCR beads, Quickprep™ total RNA extraction kit, GFX™ DNA purification kit, primers used for PCR, and cell culture mediums were purchased from Invitrogen (Stockholm, Sweden). RNA stabilisation reagent (RNAlater™) was from Qiagen GmbH (Hilden, Germany). The western blotting kit was from Amersham Biosciences (Uppsala, Sweden). All other chemical agents were purchased from Sigma Co. (Stockholm, Sweden). Human liver biopsy samples were obtained from the University Hospitals in Lund and Malmö, under the ethical permission issued by the Regional Human Ethics committee and the consents of each patient.

### Methods

#### Cell culture

HepG2 and COS-7 cells were cultured in DMEM medium with 2 mM glutamine, 4500 mg l^−1^ glucose, 100 IU ml^−1^ penicillin, 10 *μ*g ml^−1^ streptomycin and 10% heat-inactivated foetal calf serum as described (Duan *et al*, 2003). Culturing HepG2 cells under polarising conditions was performed on the insert filters (3.0 *μ*m, Corning BV, The Netherlands) as described previously (Wu *et al*, 2004a). After culturing, the cells were scraped and centrifuged by 3000 r.c.f. at 4°C for 10 min. The pellets were lysed, sonicated and centrifuged as described (Liu *et al*, 2002). The activity of alk-SMase and the protein content were determined.

#### Amplification and cloning of alk-SMase cDNA from HepG2 cells

HepG2 cells were suspended in RNAlater solution. Total RNA was extracted by Total RNA Extraction kit (Amersham Biosciences) and reversely transcripted to cDNA by a Thermoscript RT–PCR System (Invitrogen). Alk-SMase cDNA was amplified by PCR using sense primer 5′tcggtaccgaaagcatgagaggcccggccgtcctc3′ and antisense primer 5′tagcggccgcctgcgacctcagacagaagaat3′ according to the cDNA sequence of *alk-SMase* gene (GenBank AY230663) with the cDNAs as templates. The PCR products were isolated by 1% agarose gel electrophoresis and each band was purified by GFX DNA purification kit. The products were digested with *Kpn*I/*Not*I and constructed into *Kpn*I and *Not*I sites of pcDNA4/TO/myc-His plasmid. The cDNA inserts were sequenced by Cybergene (Huddinge, Sweden) using sense 5′cgcaaatgggcggtaggcgtg3′ and antisense 5′tagaaggcacagtcgagg3′primers of the vector and an alk-SMase primer 5′ggtggtgggacaacggca3′ from the site 349–367, based on the gene sequence of human alk-SMase (Duan *et al*, 2003) (GenBank AY230663).

#### Genomic alk-SMase DNA extraction and sequence

The genomic DNA of HepG2 cells was examined as described previously (Wu *et al*, 2004a). The cells were lysed and the lysate were used as template. The genomic DNA of alk-SMase gene from exon 3 to exon 5 was amplified by PCR using sense 5′cacggcatgacgaccgtggacaaac3′ and antisense 5′tagcggccgcctgcgacctcagacagaagaat3′ primers according to the genomic sequence of *ENPP7* in GenBank (NM_178543). The 2.6 kb PCR product was sequenced by Cybergene with sense 5′cacggcatgacgaccgtggacaaac3′ and 5′gccttccactacgccaacaa3′, and antisense 5′tagcggccgcctgcgacctcagacagaagaat3′, and 5′tgcatgaggtgctcgtgaga3′ primers.

#### Transient expression

COS-7 cells, which do not express endogenous alk-SMase, were transfected with 4 *μ*g of the constructed plasmids with the PCR products identified from HepG2 cells or the clone AK126250 by the lipofectamine as described previously (Duan *et al*, 2003). Control cells were transfected with the mock plasmid in the same way as the transfected cells. The cells were then cultured for 48 h, scraped and lysed as described (Duan *et al*, 2003). The activities of alk-SMase in the cell-free extracts were determined. The efficiency of the transfection was monitored by either western blotting or the simultaneous transfection of *Lac-Z* gene followed by *β*-galactosidase analysis.

#### Liver sample preparation

For alk-SMase assay, human liver biopsy samples were homogenised in 50 mM Tris–HCl buffer containing 2 mM EDTA, 1 mM PMSF, 1 mM benzamidine, 0.5 mMdithiotheritol and 2.5 mg ml^−1^ bile salts, followed by sonication for 10 s. The sonicates were centrifuged at 10 000 r.c.f. for 10 min and the supernatants were saved for biochemical analysis.

#### Determination of SMase, galactosidase and proteins

The activities of acid, neutral and alk-SMase were determined as described, using choline-labelled SM ([^14^C-SM]) as substrate in different assay buffers (Duan and Nilsson, 2000; Liu *et al*, 2006). The activity of *β*-galactosidase in COS-7 cells after transfection was assayed by a *β*-gal assay kit (Invitrogen), using ortho-nitrophenyl-*β*-galactopyranoside as substrate (Wu *et al*, 2004a). The protein was analysed by a kit from Bio-Rad (Sundbyberg, Sweden) using bovine albumin as a standard.

#### Western blot

Western blot for alk-SMase was performed as described (Duan *et al*, 2003). In brief, the cell lysate containing 50 *μ*g cellular proteins was subjected to 10% SDS–polyacrylamide gel electrophoresis and then transferred to a nitrocellulose membrane electrophoretically. After blocking, the membrane was probed with anti-human SMase antibody (1 : 2500) and then reacted with rabbit IgG antibody (1 : 50 000) conjugated with horseradish peroxidase. The alk-SMase bands were identified by ECL advanced reagents and the emitted light was recorded on Kodak X-ray film. When necessary, the membranes were then stripped and reprobed with anti-actin antibody as a loading control.

## RESULTS

### Expression of alk-SMase in HepG2 liver cancer cells

**Figure 1:**
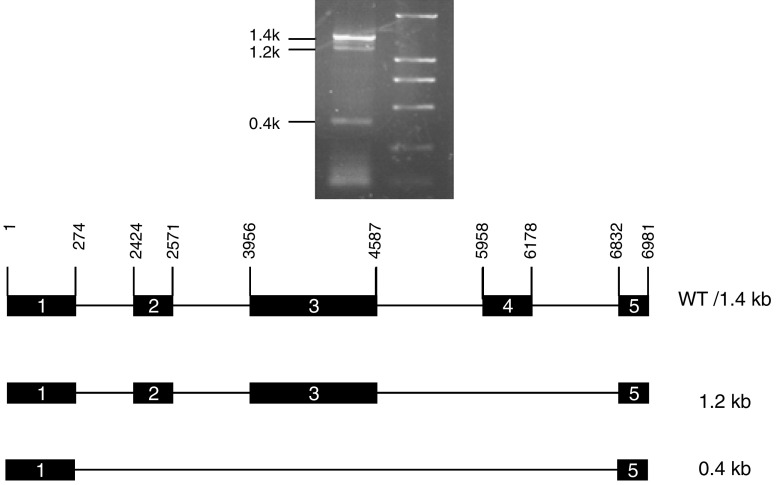
Identification of *alk-SMase* cDNA in HepG2 cells. Total RNA was extracted from HepG2 cells and *alk-SMase* cDNA was amplified by RT–PCR. The PCR products were visualised by 1% agarose gel electrophoresis (upper panel). The lower panel shows the exons found in the PCR products based on DNA sequence data. The black boxes indicate the translated exon and the order of the exon is indicated.

**Figure 2:**
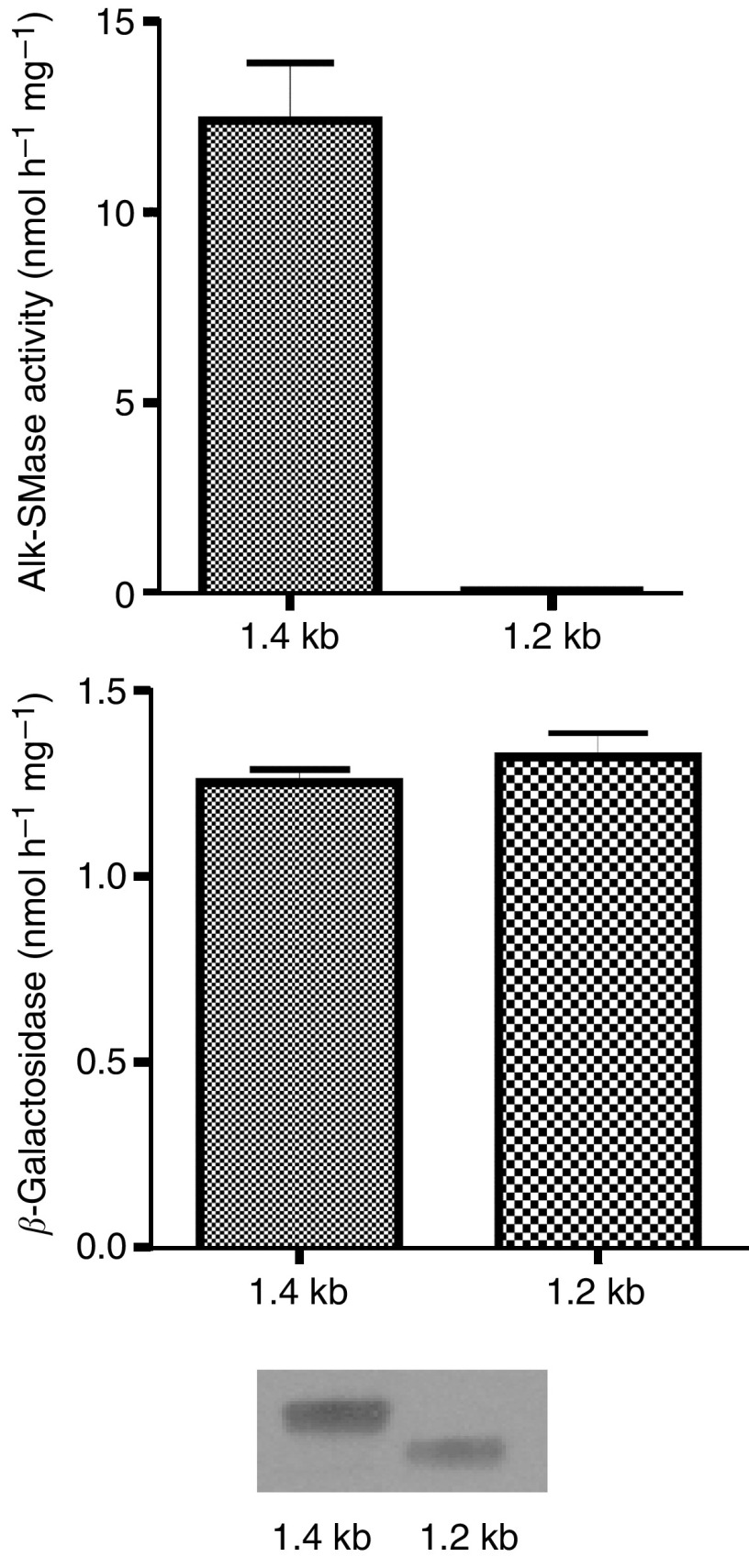
Transient expression of the wild-type and the mutant form of *alk-SMase* mRNA in COS-7 cells. The 1.4 and 1.2 kb transcript found in HepG2 cells were purified, cloned and constructed in pcDNA4/TO/myc-His. After transfecting COS-7 cells for 48 h culture, the cells were lysed and the activities of alk-SMase (upper panel) and *β*-galactosidase (middle panel) were determined. Western blot of the cell lysate for alk-SMase was performed (bottom panel). Results for activity assay were mean±s.e.m. from three separate experiments.

After RT–PCR in total RNA isolated from HepG2 liver cancer cells, three products of 1.4, 1.2 and 0.4 kb were identified by agarose gel electrophoresis (Figure 1, upper panel). Each product was then purified, cloned and sequenced. The results are shown in the lower panel of Figure 1. The 1.4 kb form turned out to be a wild-type cDNA of *alk-SMase*, which contains five translated exons as indicated. The 1.2 kb form is a product with exon 4 truncated. The 0.4 kb form is only a link of exons 1 and 5. Both 1.4 and 1.2 kb forms were then subcloned and transiently expressed in COS-7 cells. Expression of the 1.4 kb cDNA demonstrated high alk-SMase activity, whereas expression of the mutant 1.2 kb form showed no alk-SMase activity (Figure 2). The similar activity of *β*-galactosidase in both cases (Figure 2, middle panel) as well as the Western blot (Figure 2, bottom panel) confirms that the abolishment of alk-SMase activity of 1.2 kb was not caused by the insufficient transfection.

To further address whether the formation of the 1.2 kb form is caused by genomic mutation, the genomic domain between exons 3 and 5 in HepG2 cells was extracted by PCR and sequenced. No mutation at genomic level was identified (data not shown). The 1.2 kb form was therefore caused by an alternative splicing.

**Figure 3:**
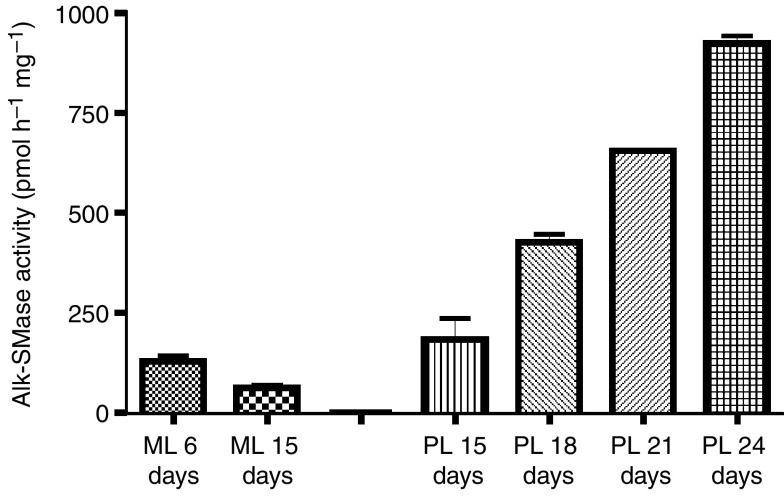
Expression of alk-SMase in HepG2 cells. HepG2 cells were cultured in both monolayer for 6 (80% confluence) and 15 days, and in polarised conditions for 15, 18, 21 and 24 days. The cell-free extracts were prepared and alk-SMase activity in the cell lysate was determined. ML, monolayer; PL, polarised. The days of culturing were indicated. Results are mean±s.e.m. form duplicate determinations in three separate experiments.

Since both wild-type and mutant *alk-SMase* mRNAs were identified in HepG2 cells, the expression of the wild-type enzyme in the cells was examined in both monolayer and polarised conditions. As shown in Figure 3, the activity of alk-SMase was low in monolayer cells and was maximal at about 80% confluence (6 days) and then declined when the culturing was continued. However, in the cells cultured in polarised conditions, the alk-SMase activity was significantly increased with the time. The results indicate that the expression of the wild-type alk-SMase is associated with differentiation.

**Figure 4:**
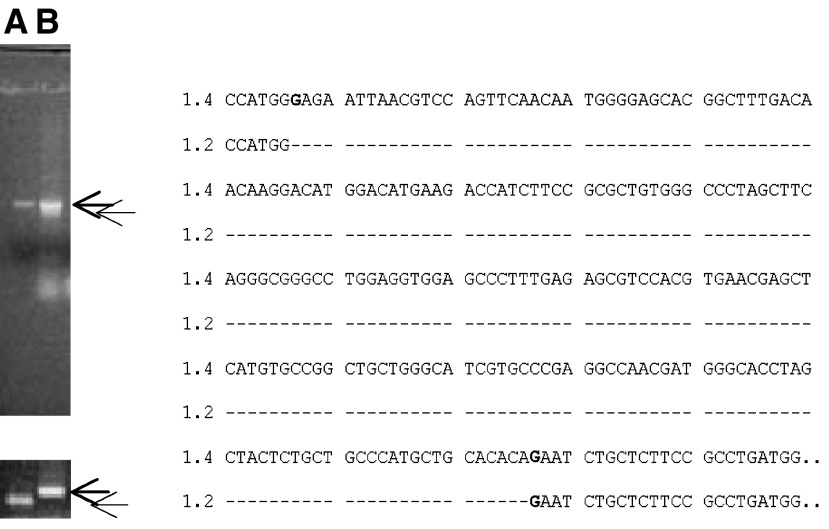
Identification of *alk-SMase* cDNA in one hepatoma tissue from a patient with autoimmune hepatitis. The *alk-SMase* cDNA was amplified by PCR using the total cDNA isolated from the tissue as a template. The PCR products were visualised by 1% agarose electrophoresis (left panel). The two PCR products were further purified and amplified by second PCR (lower left panel). The difference in cDNA sequence is shown on the right panel, which shows the absence of exon 4. (A) cDNA of wild-type *alk-SMase* as a template. (B) cDNA of the hepatoma as a template. The two arrows indicate the 1.4 and 1.2 kb forms identified.

How often the alternative 1.2 kb mRNA occurs in different types of liver cancer is unknown, since the answer to this question requires studies of a large number of liver tumours, which are not very common in Sweden. In a preliminary study, we performed similar PCR using a cDNA template isolated from a hepatoma tissue of a patient with autoimmune hepatitis. Two PCR products with the size of 1.4 and 1.2 kb were identified (Figure 4, upper left). The two products were then purified, amplified by PCR (Figure 4, lower left panel) and sequenced. We found that the 1.4 kb is the wild-type mRNA, whereas the 1.2 kb is identical to that found in HepG2 cells, without exon 4 (Figure 4, right panel). The results indicate that the 1.2 kb aberrant form can be identified in human liver cancer tissue.

### Expression of a ‘big’ alk-SMase in liver tumour

**Figure 5:**
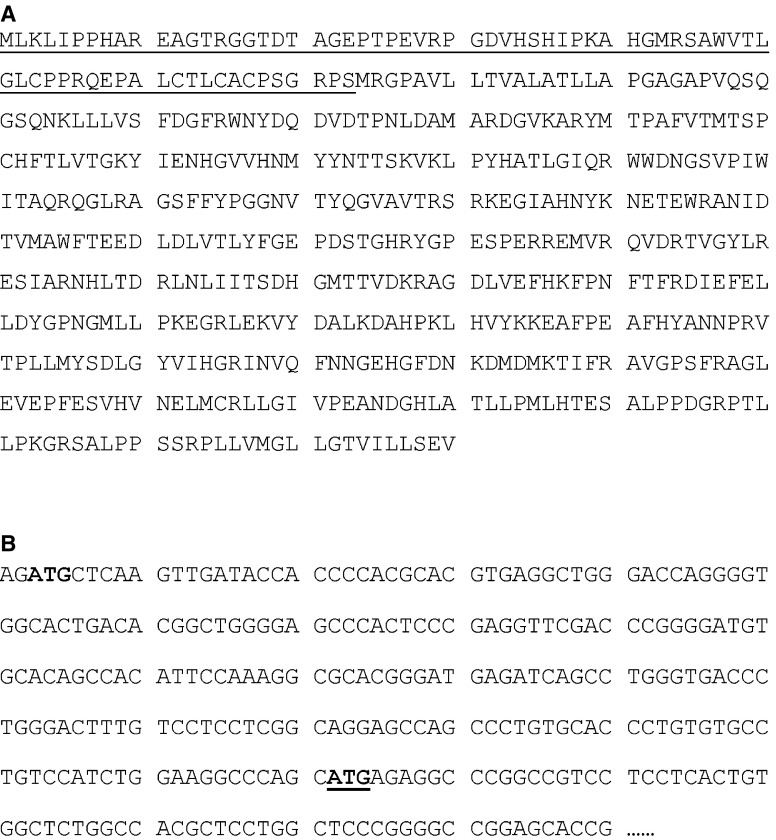
The amino-acid and cDNA sequences of AK126250 clone identified in liver tumour. The panel **A** shows the full length of amino-acid sequence of AK126250. The underlined sequence indicates the N-terminal tag that links to the wild-type alk-SMase. The panel **B** displays part of the cDNA sequence of AK126250. The start codon of the protein is in bold, and that of the wild-type *alk-SMase* is in bold and underlined.

**Figure 6:**
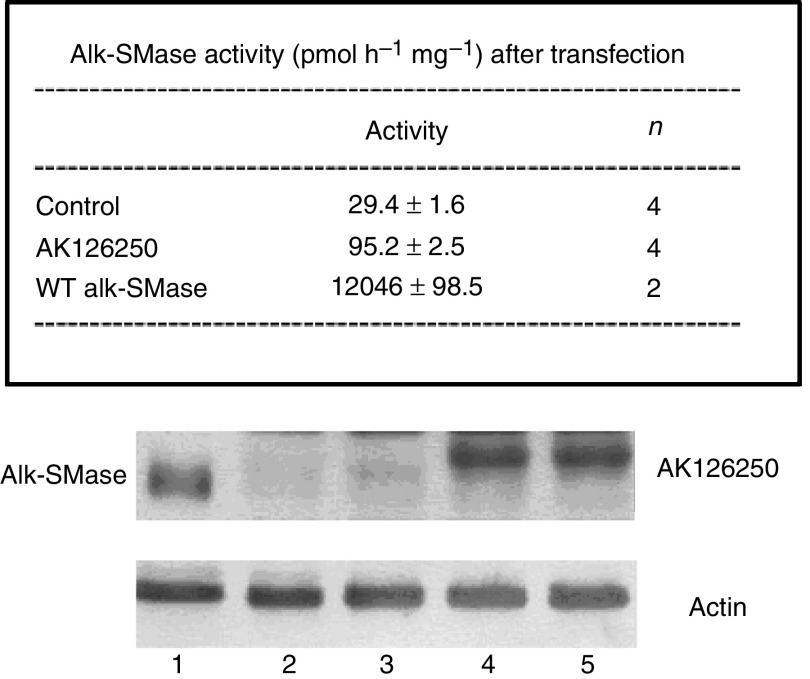
Expression of cDNA of wild-type *alk-SMase* and the AK126250 in COS-7 cells. COS-7 cells were transfected with the wild-type and AK126250 cDNA. The alk-SMase activities in the lysates 24 h after transfection were determined and the results are shown in the box of the upper panel. The expressed wild-type alk-SMase and the AK126250 proteins were shown by Western blot in the lower panel. Lane 1: wild-type alk-SMase expressed in COS-7 cells. Lanes 2 and 3: control COS-7 cells. Lanes 4 and 5: AK126250 expressed in COS-7 cells. A loading control by probing the membrane with anti-actin was shown below.

**Figure 7:**
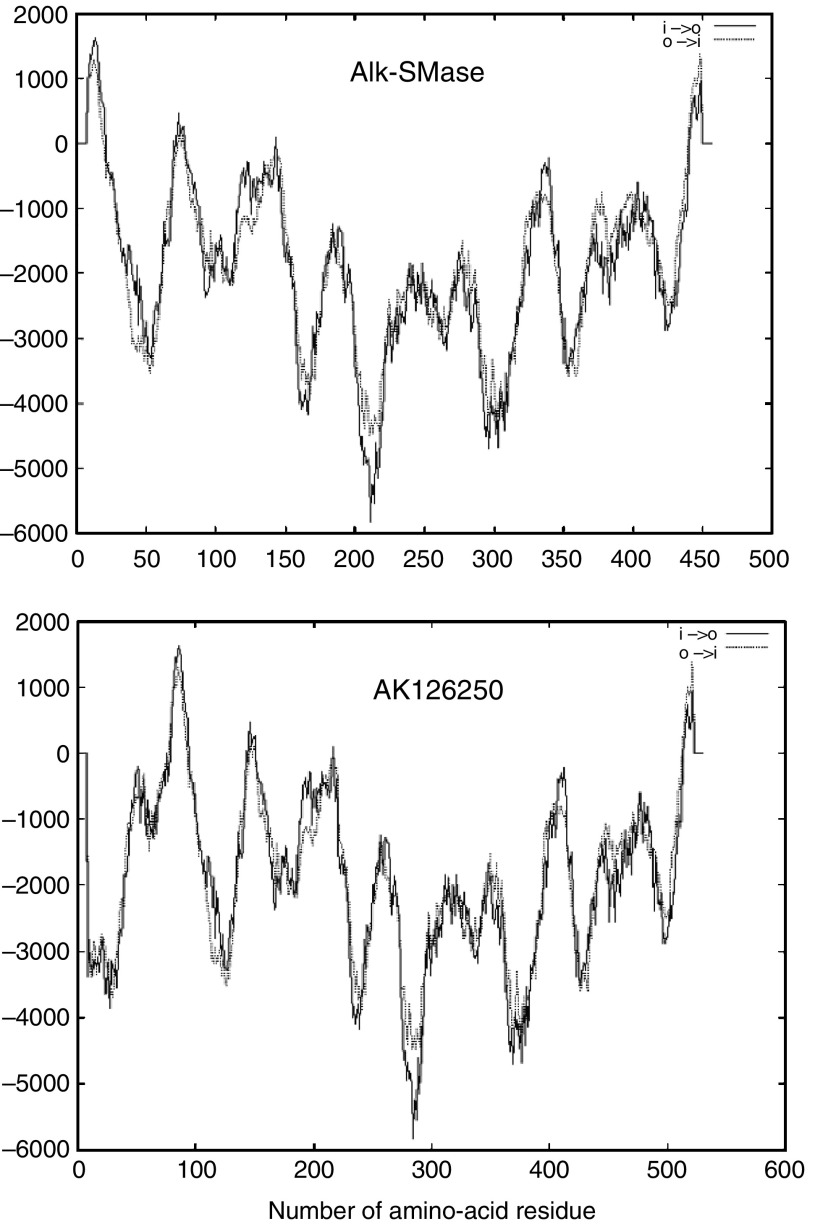
Comparison of the TM-pred analysis of N-terminal transmembrane domains of wild-type alk-SMase and AK126250 protein. The *X*-axis is the amino-acid sequences of the proteins and the *Y*-axis is the hydrophobicity of the amino-acid residues.

By FIS (full-length insert sequence) searching in the GenBank, we found a cDNA clone (GenBank AK126250) with 2031 bp that encodes full length of alk-SMase plus a 73-amino-acid tag linked to the N terminus of the enzyme (Figure 5, upper panel). The clone for this ‘big’ alk-SMase was identified from a human liver tumour. Looking at the genomic sequence, we found that the big alk-SMase is translated by an alternative start codon, which is located upstream of the wild-type mRNA of *alk-SMase* (Figure 5, lower panel). To investigate whether this protein has alk-SMase activity, the cDNA clone was purchased and expressed in COS-7 cells. As shown in the box of Figure 6, the alk-SMase activity in the cells transfected with AK126250 was three-fold higher than that in control cells but still much lower than that in the cells transfected with wild-type *alk-SMase*. Western blot clearly showed that the AK126250 protein had been expressed to similar level as the wild-type alk-SMase cDNA (Figure 6, lower panel). Since the N terminus of wild-type alk-SMase contains a transmembrane domain that serves as a signal peptide, TM-pred analysis was performed for AK126250 protein. As shown in Figure 7, linking the 73 residues at the N-terminal changes the hydrophobic feature of the enzyme. The big alk-SMase no longer has a signal peptide at the N terminus.

We also examined whether the big alk-SMase is expressed in HepG2 cells by RT–PCR using new sense primer 5′tcggtaccgaaagcatgctcaagttgataccac3′ and the wild-type antisense primer according to the sequence of the big enzyme. No PCR product with correct size was identified, indicating that this type of alk-SMase is not expressed in HepG2 cells (data not shown).

### Alk-SMase activity in liver diseases

For addressing a question whether alk-SMase activity is detectable in human liver biopsies, three types of SMase were analysed in 30 biopsy samples. The results with the diagnosis are shown in Table 1. The activity of alk-SMase was detectable in liver biopsies and was high comparing with acid and neutral SMase. Although not conclusive due to the number of the samples, alk-SMase activity appeared particularly high in cholestatic liver disease excluding primary sclerosing cholangitis (PSC) (2.83±1.4 nmol h^−1^ mg^−1^) and low in steatosis (0.41±0.1 nmol h^−1^ mg^−1^) and PSC (0.72±0.37 nmol h^−1^ mg^−1^). The activity did not correlate with most biochemical examinations, except that a positive correlation with *α*-antitrypsin was identified (*r*^2^=0.1658, *P*=0.0350).

## DISCUSSION

Alk-SMase is specifically expressed in the intestinal tract and human liver. The clinical implications of the enzyme in intestinal tumours have been a topic under the last decade. The accumulating evidence indicates that, in the intestinal tract, alk-SMase may prevent colonic tumorigenesis. First, the enzyme generates antiproliferative and proapoptotic lipid messenger ceramide (Duan *et al*, 2003; Hertervig *et al*, 2003). Second, the enzyme inhibits proliferation of human colon cancer cells (Hertervig *et al*, 2003). Third, the enzyme activity is reduced in both longstanding ulcerative colitis and colonic tumorigenesis (Hertervig *et al*, 1997, 1999; Sjöqvist *et al*, 2002). And finally, mutation has been identified in colon cancer cells, which abolishes the enzyme activity (Wu *et al*, 2004a).

Human liver is the only organ except the intestine that expresses alk-SMase. It is both a site of primary tumours and the most common site of colon cancer metastasis. It is well-known that PSC increases the risk of hepatobiliary cancer (Yang *et al*, 2001; Ohata *et al*, 2003; Lazaridis and Gores, 2006) as well as colon cancer associated with colitis (Broome and Bergquist, 2006). The disease is treated with ursodeoxycholic acid to decrease the risk of colorectal cancer (Pardi *et al*, 2003) and ursodeoxycholic acid has been shown to induce expression alk-SMase in both animal models and cell culture studies (Cheng *et al*, 1999; Liu *et al*, 2006). We therefore raised a question whether this ectoenzyme may also be an antitumour factor both in development of primary hepatic and biliary carcinomas and in the regulation of metastatic tumour growth. To answer the question, it is important to study the expression of the enzyme associated with liver cancer and precancerous diseases.

In this study, we showed that HepG2 liver cancer cells have both wild-type (1.4 kb) and mutant forms (1.2 and 0.4 kb) of *alk-SMase* transcript. The identification of the 1.2 kb form may have special importance, because it is an inactive form and it resembles the mutation previously identified in HT29 colon cancer cells (Wu *et al*, 2004a). Although the mutations found in HepG2 and HT29 cells are not identical, both mutations involve a deletion of exon 4. We previously showed that the amino acid His at 353 position in exon 4 is critical for the enzyme function, and site mutation of H353 abolishes the enzyme activity (Wu *et al*, 2004a). H353 in fact participates in the formation of two metal coordinate sites, and recent studies showed that metal ion sites in NPP family may play important roles in substrate binding (Zalatan *et al*, 2006). It is therefore expected that the protein encoded by the 1.2 kb form showed no alk-SMase activity. The similar exon 4 deletion in both liver and colon cancer cells casts light on the close link of colon and liver in tumorigenesis. Unlike HT29 colon cancer cells, HepG2 cells also express the wild-type alk-SMase. The expression rate is low under monolayer conditions, but increased when the cells are under polarising, that is, differentiating conditions. The finding is in agreement with our previous study on Caco-2 cells (Wu *et al*, 2004a). Thus, the factors that inhibit the differentiation of liver cells may suppress the expression of alk-SMase.

The present study also characterised another form of alk-SMase registered in GenBank, which was identified in a liver tumour. The cDNA clone encodes the full length of the enzyme plus 73-amino-acid residues linking to the N terminus. It is translated from an alternative starting codon upstream of the wild-type mRNA. Although transient expression showed some activity of the big alk-SMase, but the activity was much lower than the wild-type enzyme. The reduction may be caused by the change of the hydrophobicity of alk-SMase at the N terminus, leading to loss of a signal peptide, which regulates the traffic of the enzyme to endoplasmic reticulum for glycosylation and to the plasma membrane for secretion (Wu *et al*, 2005b). Without the signal peptide, the enzyme may not be properly transported and glycosylated. Normal transportation and glycosylation are important factors for full activity of the enzyme (Wu *et al*, 2005b).

By measuring acid, neutral and alkaline SMase activities in human liver biopsy samples, we showed that alk-SMase activity is detectable in human liver biopsies. The activity is high and at least equal to that of acid SMase, which is known to be highly expressed in the liver (Spence *et al*, 1979). The biopsy samples examined were taken from patients with different liver diseases. The number of observations was too small to conclusively identify differences in alk-SMase expression among different diseases. However, relatively low activities were indicated in steatosis and PSC, which increases risk for hepatocellular carcinoma (Yang *et al*, 2001; Ohata *et al*, 2003) and cholangiocarcinoma (Lazaridis and Gores, 2006), respectively. The finding may indicate an early reduction of the enzyme activity in precancerous diseases in the liver. Alk-SMase activity in general did not significantly correlate with many of the routine liver tests, but positively with the level of *α*-antitrypsin. The significance of this finding is unknown. Since liver cells express trypsin-like protein (Leytus *et al*, 1988) and trypsin has been shown to dissociate alk-SMase from the intestinal mucosa (Wu *et al*, 2004b), anti-trypsin may prevent alk-SMase from release and thus increase its levels.

By examining a cDNA sample isolated from a biopsy of hepatocellular carcinoma, we did find similar coexistence of 1.4 and 1.2 kb mRNA of alk-SMase in the tissue, indicating that such mutation does occur in human liver cancer tissues. So far we have, however, not had access to enough human liver tumours to provide data on the frequency of different alk-SMase mutations in such tumours. Obviously this is an important issue for further studies.

In conclusion, our results identified mutations of alk-SMase in liver tumorigenesis, resulting in significant reduction of the enzyme activity. The identification of similar deletion of exon 4 in colon and liver cancers may give insight into the frequent liver metastasis of colon cancer. Further investigations with larger number of tissue samples should focus on type and frequency of the mutations and their pathological implications in both cancer and precancerous liver diseases.

## Figures and Tables

**Table 1:** SMase activity (nmol h^−1^ mg^−1^) in liver biopsy samples

**Patient**	**Age (years)**	**Sex**	**A-SMase**	**N-SMase**	**Alk-SMase**	**Diagnosis**
1	70	F	2.253	1.574	2.143	Cholestatic, not PBC
2	72	M	4.412	3.462	5.527	Cholestatic, not PBC
3	64	F	1.136	1.013	0.824	Cholestatic, not PBC
4	30	M	1.293	2.174	0.308	Steatosis
5	73	M	1.105	1.571	0.745	Steatosis
6	69	M	0.825	0.316	0.431	Steatosis
7	60	M	0.837	0.375	0.439	Steatosis
8	67	M	1.356	0.389	0.105	Steatosis, mild hepatitis
9	63	F	1.926	1.181	1.415	PSC
10	31	M	0.758	0.657	0.562	PSC
11	42	M	1.009	1.582	0.174	PSC
12	47	M	0.621	0.897	1.093	NASH
13	74	M	2.372	1.015	1.120	NASH
14	72	F	1.941	2.921	4.586	Toxic, including drug not alcohol
15	79	F	1.982	1.690	1.817	Toxic, including drug not alcohol
16	73	F	1.379	0.964	1.249	PBC
17	74	F	0.883	1.296	1.593	PBC
18	85	M	2.511	1.246	1.778	Cryptogenic cirrhosis
19	83	M	1.310	1.818	2.039	Cryptogenic cirrhosis
20	65	M	1.122	0.396	0.456	Cryptogenic hepatitis
21	53	F	0.860	0.776	0.862	Alcoholic cirrhosis
22	54	F	2.768	1.387	2.011	Alcoholic cirrhosis
23	69	F	0.977	2.570	3.633	Alcoholic hepatitis
24	33	M	0.957	1.000	1.378	Autoimmune hepatitis
25	25	F	1.258	0.851	0.439	Autoimmune hepatitis
26	41	F	0.339	0.574	0.513	Haemochromatosis
27	50	M	0.772	0.413	0.418	Haemochromatosis
28	37	M	0.754	0.541	0.424	Chronic hepatitis C
29	51	F	0.750	1.519	2.189	Viral infection
30	90	F	1.155	0.551	0.448	Systemic disease

A-SMase=acid sphingomyelinase; Alk-SMase=alkaline sphingomyelinase; F=female; M=male; PBS=primary biliary cirrhosis; PSC=primary sclerosing cholangitis; NASH=non-alcoholic steatohepatitis; N-SMase=neutral sphingomyelinase.
